# Impact of COVID-19 on Maternal Health Service Uptake and Perinatal Outcomes in Sub-Saharan Africa: A Systematic Review

**DOI:** 10.3390/ijerph21091188

**Published:** 2024-09-06

**Authors:** Zemenu Yohannes Kassa, Vanessa Scarf, Sabera Turkmani, Deborah Fox

**Affiliations:** 1 Collective for Midwifery, Child and Family Health, Faculty of Health, University of Technology Sydney, P.O. Box 123, Broadway, NSW 2007, Australia; vanessa.scarf@uts.edu.au (V.S.); sabera.turkmani@uts.edu.au (S.T.); deborah.fox@uts.edu.au (D.F.); 2 College of Medicine and Health Sciences, Hawassa University, Hawassa P.O. Box 1560, Ethiopia

**Keywords:** COVID-19, maternal health, health service, Sub-Saharan Africa

## Abstract

Coronavirus 2019 (COVID-19) is a major global public health threat that has impeded health infrastructures in low- and middle-income countries. This systematic review examines the impact of COVID-19 on maternal health service uptake and perinatal outcomes in Sub-Saharan Africa. We searched four databases in August 2020 and updated the search on 22 December 2023: PubMed/MEDLINE, CINAHL, Maternity and Infant Care, and EMBASE. Data extraction was performed using a standardised Joana Briggs Institute data extraction format for the eligibility of articles, and any discrepancies were solved through discussion and consensus. This systematic review includes 36 studies that met the inclusion criteria. Antenatal care attendance and institutional childbirth significantly decreased during the COVID-19 pandemic, and home births increased. Fear of contracting the virus, a lack of transport, a shortage of logistic supplies, a lack of personal protective equipment, lockdown policies, economic and food security, stigmatisation of sick persons, long waiting times in the hospital, and health system weakness were barriers to accessing maternity care. The findings of this review showed a significant decrease in antenatal care attendance and institutional birth during the COVID-19 pandemic. Based on our findings, we recommend that stakeholders ensure the availability of essential medical supplies in the hospital.

## 1. Introduction

COVID-19 is a highly contagious viral pneumonia caused by severe acute respiratory syndrome coronavirus 2 (SARS-CoV-2) [1]. This is a rapidly spreading virus with cases found worldwide since its first identification in Wuhan, China, in December 2019 [2,3]. The pandemic has caused a significant global public health problem that has interrupted health system service delivery and infrastructure, most notably in low- and middle-income countries (LMICs) [4].

A single disease, COVID-19, has shown how the daily lives of infected and non-infected people can be affected in many ways [5,6]. One of its effects was the restriction of non-essential movement between local areas and abroad for more than a year. Additionally, the world focused on preventing the spread of COVID-19, and the subsequent loss of key workers through illness, death, or self-isolation following contact with infected people [7] has impacted healthcare systems across LMICs.

Pregnant women have experienced the direct and indirect impacts of COVID-19 on their health, and the pandemic has continued to negatively impact many pregnant women and their offspring [8]. The physiological, immunological, anatomical, and hormonal changes that occur during pregnancy [9] leave women more vulnerable than the general population to emerging infectious diseases. Past infectious diseases such as severe acute respiratory syndrome (SARS), Middle East respiratory syndrome (MERS), Ebola virus disease (EVD), H1N1 pandemic influenza, and Zika virus [10] have caused adverse pregnancy outcomes including renal failure, sepsis, disseminated intravascular coagulation (DIC), death [11], spontaneous abortion [12], preterm birth, stillbirth [13], intrauterine growth restriction [14], preeclampsia [15], haemorrhage, and microcephaly [16,17].

A pandemic affects the efficiency of the healthcare system in several ways when it comes to providing maternal and perinatal care [18]. Limitations of infrastructure, human resources, supply chains, and financial resources compromise health system functions, making them less able to offer services and implement rapid adaptations in antenatal care (ANC) and intrapartum care access, uptake, and provision [19]. Already limited resources were shifted in resource-scarce countries to accommodate COVID-19 prevention and treatment [20], and non-emergency services may have been discontinued, affecting access and uptake of routine activities [21]. Barriers to accessing care and services include movement restrictions, a lack of public transport, and fear of contracting COVID-19 in health facilities in LMICs, including Sub-Saharan Africa [20,22].

According to initial findings, ANC and intrapartum care uptake in LMICs was disrupted [23], and maternal and newborn mortality rates increased [24,25]. This pandemic has the potential to reverse the remarkable achievements made in reducing maternal and neonatal morbidity and mortality in resource-limited countries over the past two decades [26]. With these great strides, ANC and intrapartum care uptake and rates of maternal and neonatal mortality had steadily improved in low-income countries. It is challenging for these countries to attain the Sustainable Development Goals (SDGs) [27], and extraordinary strategies have been employed to reach the desired level; for maternal mortality, a ratio of <70 maternal deaths per 100,000 live births and, for neonatal mortality, <12 neonatal deaths per 1000 live births are the Sustainable Development Goals (SDG 3.1) in every country by 2030.

Several studies have nuanced the impact of COVID-19 on maternal healthcare information in Sub-Saharan Africa. However, there is a paucity of comprehensive reviews addressing the impact of COVID-19 on maternal health service uptake and perinatal outcomes. Conducting a critical review and appraising empirical studies to synthesise their findings is crucial for providing stakeholders and policymakers with the necessary information to develop strategies aimed at achieving SDG 3.1 and preparing for future maternal health challenges, offering a valuable lesson learned. This understanding is pivotal for preserving the progress made over the past two decades and ensuring effective measures are in place to mitigate the impact of similar disruptions in the future. Therefore, this systematic review synthesises the impact of the COVID-19 pandemic on maternal health service uptake and barriers to access and perinatal outcomes in Sub-Saharan Africa.

## 2. Methods

### 2.1. Information Sources and Search Strategy

**Figure 1:**
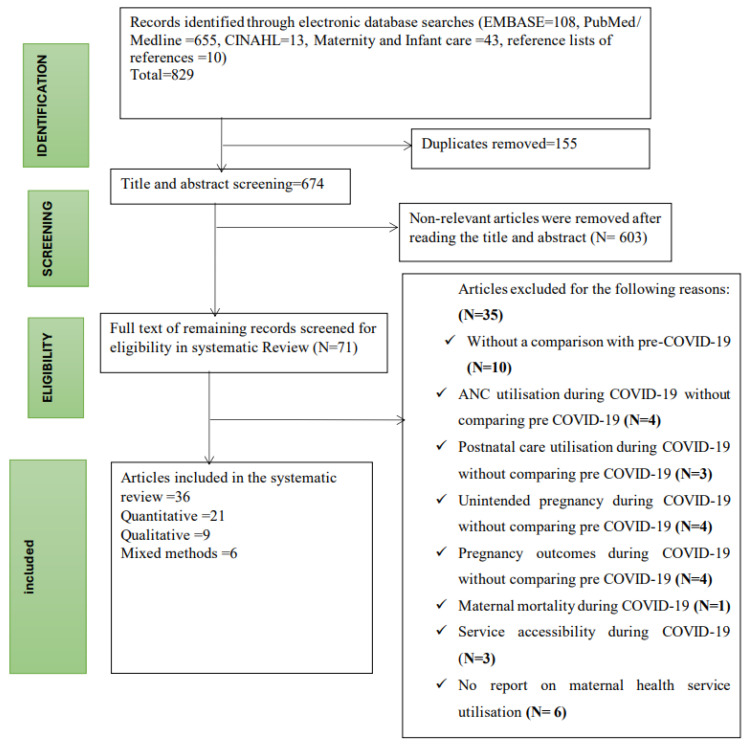
PRISMA (flow chart of study selection for a systematic review of the effect of COVID-19 on maternal and perinatal care in Sub-Saharan Africa).

Four databases were searched in August 2020, and the search was updated on 22 December 2023: PubMed/MEDLINE (Ovid), CINAHL (EBSCO hosted platform), Maternity and Infant Care (Ovid), and EMBASE (Ovid). Specifically, the focus was on articles that assessed the impact of COVID-19 on maternal health service uptake. For a search strategy, the combination of the following medical heading subject (MeSH) terms and keywords were used: “Maternal health service” OR “Reproductive health service” OR “Antenatal care” OR “Obstetrics health service” OR “Maternal and newborn health service” OR “Maternal primary care” OR “Postnatal care” OR “Maternal Health” OR “Maternal-child health services” AND “COVID-19” OR “SARS-CoV-2” AND “Utilisation/utilization” AND “Angola” OR “Benin” OR “Botswana” OR “Burkina Faso” OR “Burundi” OR “Cameroon” OR “Cape Verde” OR “Central African Republic” OR “Chad” OR “Comoros” OR “Republic of the Congo” OR “The Democratic Republic of the Congo” OR “Cote d’Ivoire” OR “Djibouti” OR “Equatorial Guinea” OR “Eritrea” OR “Ethiopia” OR “Gabon” OR “Gambia” OR “Ghana” OR “Guinea” OR “Guinea-Bissau” OR “Kenya” OR “Liberia” OR “Madagascar” OR “Malawi” OR “Mali” OR “Mauritania” OR “Mauritius” OR “Mozambique” OR “Namibia” OR “Niger” OR “Nigeria” OR “Rwanda” OR “Sao Tome and Principe” OR “Senegal” OR “Seychelles” OR “Sierra Leone” OR “Somalia” OR “South Africa” OR “South Sudan” OR “Sudan” OR “Swaziland” OR “Tanzania” OR “Togo” OR “Uganda” OR “Zambia” OR “Zimbabwe” OR Sub Saharan Africa”. Hand searches of the reference lists were carried out to identify other potential articles of interest. The included studies were limited to the English language and human studies only. The protocol was registered with the PROSPERO International Register of Systematic Reviews (https://www.crd.york.ac.uk/PROSPERO) on 15 October 2020 and updated on 19 July 2024 (CRD42020208198). This section follows the Preferred Reporting Items for Systematic Reviews and Meta-Analysis (PRISMA) checklist (Supplementary Materials) [28] to present findings on the effect of COVID-19 on maternal health service utilisation (Figure 1).

### 2.2. Selection of Studies and Data Extraction

One reviewer (ZYK) ran the search strategy across the relevant databases and exported it to Endnote 20 to remove duplicate articles in the review. Data were extracted by four authors (ZYK, VS, ST, and DF) based on the article title and abstracts for eligibility. These authors (ZYK, VS, ST, and DF) also extracted all the relevant data using a standardised Joanna Briggs Institute (JBI) data extraction format for the eligibility of articles, and any discrepancies were solved through discussion and consensus. The author’s name, publication year, purpose of the study, country, study design, response rate, utilisation of maternal health services, and outcome measures were extracted.

### 2.3. Inclusion and Exclusion Criteria

Studies assessing the impact of COVID-19 on ANC attendance, institutional birth, and pregnancy outcomes were eligible for inclusion in this systematic review. The included studies were those that compared pre- and during COVID-19 maternal service uptake and were conducted with the following study designs: case series, observational studies (cross-sectional, pre-post, and cohort (prospective and retrospective studies)), and qualitative studies regardless of their study settings. Editorial letters, commentaries, review articles, articles that did not compare maternal health service uptake pre- and during COVID-19, case reports with incomplete information, modelling studies, magazine articles, and personal opinions were excluded. The most recent article was used when multiple publications of the same data were found.

### 2.4. Assessment of Quality of Included Studies

The data quality was assessed using the Joanna Briggs Institute (JBI) [29] critical appraisal checklist for simple prevalence, containing nine checklist items and ten items for qualitative studies. This tool lists the following criteria to assess the quality of studies: the sample frame must be appropriate to address the target population; the study participants must be sampled in an appropriate way; the sample size must be adequate; the study subjects and the setting must be described in detail; the data analysis must be conducted with sufficient coverage of the identified sample; valid methods must be used for the identification of the condition; the condition must be measured in a standard, reliable way for all participants; an appropriate statistical analysis must be utilised; and the response rate must be adequate; and the low response rate must be managed appropriately [29]. Studies that scored below five out of these nine points were considered to be of low quality, while those with scores above five were deemed sufficient for inclusion in this review.

### 2.5. Data Analysis

A narrative synthesis was used to present the findings. The findings were presented by highlighting the decrease in the number of women attending ANC, the impact of COVID-19 on institutional births and mode of birth, complications during childbirth, and barriers to the uptake of maternity care during the pandemic.

## 3. Results

In this study, 829 studies were retrieved from four databases, 155 articles were removed due to duplication, and 603 articles were removed based on the title and abstract screening. Full-text screening of 71 articles was conducted, and studies were excluded if they did not compare and report maternal health services uptake and perinatal outcomes pre-COVID-19 and during the pandemic. Thirty-six studies were finally included in this systematic review. Figure 1 illustrates the process of screening and reviewing the articles.

### 3.1. Study Characteristics

Articles included in this systematic review were from Ethiopia (*n* = 9) [30,31,32,33,34,35,36,37,38], Kenya (*n* = 9) [38,39,40,41,42,43,44,45,46], Uganda (*n* = 6) [21,38,47,48,49], Nigeria (*n* = 3) [49,50,51], Sierra Leone (*n* = 3) [22,38,52], the Democratic Republic of Congo (DRC) (*n* = 2) [53,54], South Africa (*n* = 2) [55,56], Mozambique (*n* = 2) [57,58], Liberia (*n* = 2) [22,59], Guinea (*n* = 2) [49,60], Tanzania (*n* = 2) [38,49], Rwanda (*n* = 1) [61], Zimbabwe (*n* = 1) [62], Ghana (*n* = 1) [63], and Lesotho (*n* = 1) [22]. Nine studies had a cross-sectional study design [32,33,35,39,44,55,56,60,61], nine studies had a qualitative study design [21,34,40,41,42,46,48,50,51], seven studies had an interrupted times series design [22,38,43,45,47,53,58], six studies had a mixed methods design [36,37,49,52,57,59], two studies were retrospective cohort studies [54,62], and three studies had a pre–post study design [30,31,63] (Table 1). This study included articles that used a minimum of two months pre-COVID-19 and two months during COVID-19. The maximum data used were 12 months pre-COVID-19 and 12 months during COVID-19 to assess the impact of COVID-19 on maternal health services uptake. The study participants in this review were pregnant women, women in labour, postnatal women, healthcare providers, and policymakers. Table 1 is a summary of the included studies.

After reviewing and summarising the included studies, four main themes were identified: a decrease in the number of women attending ANC; the impact of COVID-19 on institutional births and mode of childbirth; complications during childbirth; and barriers to the uptake of maternity care. Table 2 presents the themes and the papers associated with those particular themes.

### 3.2. Decrease in the Number of Women Attending ANC during COVID-19

COVID-19 has, directly and indirectly, interrupted the health system in LMICs, particularly in Sub-Saharan Africa. Outpatient services are more affected due to the closure of non-emergency services, and ANC services were also disturbed in Sub-Saharan countries during COVID-19 [22]. Evidence showed that ANC1 uptake significantly declined by 43% (IRR: 0.57, 95%CI: 0.35 to 0.91, *p* = 0.02) in DR Congo [53] and by 25% in Liberia during the lockdowns [59]. Similarly, ANC4 declined by 28% in Liberia (Table 1). Movement restrictions, a lack of transport, increased transport fees, and closure of non-emergency services could all have contributed to the decline in ANC uptake during the lockdowns [59].

ANC1 uptake significantly decreased in the Southwest region, Ethiopia (*p* < 0.0001) [32], Rwanda (*p* = 0.042) [61], Liberia [59], Guinea [60], and Sierra Leone [22] during COVID-19. ANC4 significantly decreased in the Tigray region, Ethiopia [31], Kenya [45], Ghana [63], Liberia [59], and Guinea (*p* < 0.001) [60] during COVID-19. The reasons for these findings indicate that COVID-19 affected ANC uptake indirectly, with fear of contracting a COVID-19 infection [34], staff redeployment to COVID-19 centres [59], restriction of movement due to lockdowns, a lack of PPE [41], shortages of medications and vaccine supplies for clients [59], and a partial closure of routine services [49] being common causes of the drop in ANC uptake during COVID-19 (Table 1). In DRC, immediately post-lockdowns, ANC1 contact significantly increased by 4% (1.04, 95% CI: 1.01 to 1.07, *p* = 0.007). Meanwhile, this review showed that the rates of uptake for ANC1 and above in South Africa [56], Ethiopia [33,35,36,37], Kenya [39,43], and Mozambique [57,58] did not significantly differ during COVID-19 compared with the pre-COVID-19 period (Table 1).

### 3.3. Impact of COVID-19 on Institutional Birth and Mode of Birth

Studies showed that institutional birth rates significantly increased in the early stages of COVID-19 [31,52,53,56]. For example, the institutional birth rates significantly increased by 8.57% (*p* = 0.0001) in the Tigray region, Ethiopia [31]. Immediately post-lockdowns, institutional births significantly increased by 8% (1.08, 95% CI: 1.05 to 1.011, <0.001) in DRC [53] and by 3.7% in South Africa in 2020 [56]. However, the incidence of institutional births was not significantly altered during the pandemic lockdowns compared to pre-COVID-19 in the Amhara region, Ethiopia [33], DRC [53], and Zimbabwe [62].

Other studies in Mozambique, Sierra Leone, Guinea, Uganda, Rwanda, and Ethiopia showed that during COVID-19, institutional births significantly decreased [30,32,47,52,57,60,61]. Hospital birth rates declined by 77% in urban areas of Ethiopia, immediately after COVID-19 infections were reported there (aRRR: 0.23, 95% CI: 0.07 to0.71) [30]. Similarly, in Mozambique, hospital births significantly decreased by 4% (*p* = 0.046) [57]. At the same time, the study found that home births increased by 74% [57], and in Kenya, home births also increased during COVID-19 [40,41]. Evidence showed that women preferred home births due to fear of contracting the virus [40], delayed care, a lack of money, perceived poor quality of care during COVID-19 [34], and a lack of transportation due to lockdowns [49,51,59], leading to increased numbers of stillbirths and neonatal deaths during this period [31,32,39,47,55].

During COVID-19, caesarean section births significantly increased [31,32,39]. Caesarean section births in Ethiopia [31] substantially increased by 28.05% (*p* = 0.0040), and in Kenya [39], caesarean births increased from 14.6% to 15.8% (*p* < 0.0001) during COVID-19. However, other studies did not find a significant difference in the numbers of caesarean section births before and during COVID-19 in Mozambique [57], Sierra Leone [52], and Zimbabwe [62] (Table 1).

### 3.4. Complications during Birth

Quality ANC and intrapartum care are essential for the early identification of complications, readiness for high-risk newborns, and prompt intervention [64] that can reduce stillbirth and neonatal mortality. During COVID-19, ANC uptake and institutional birth declined in LMICs [22]. Studies in Ethiopia and Kenya [31,32,39] found that stillbirths significantly increased during COVID-19, increasing by 7.6% (*p* = 0.0062) in the Tigray region, Ethiopia [31] (Table 1). Likewise, studies in Ethiopia, Uganda, and South Africa showed that neonatal deaths significantly increased [32,47,55,56] during COVID-19: For example, in South Africa, the neonatal death rate increased by 47% (*p* = 0.025) [55]. In Uganda, the neonatal mortality rate increased by ten neonatal deaths per 1000 live births/month (IQR 2–10; *p* < 0.001) at the end of the lockdowns [47]. Neonatal death rates did not, however, significantly differ in Ethiopia and Zimbabwe [31,62] before and during COVID-19. It is important to highlight that reporting of these deaths may be confounded by methods of reporting and systemic data collection issues.

### 3.5. Barriers to the Uptake of Maternity Care

Effective strategies to improve the accessibility and availability of maternal healthcare is essential to increasing its uptake. The pandemic has indirectly encumbered and overstretched the infrastructure [4] that had previously been in place to increase the uptake of maternity care. Fear of the virus and lockdown measures to prevent the spread of COVID-19 meant that transport became more expensive and often unavailable [51], making it difficult for women to access maternity care (Table 1).

Studies conducted in Nigeria [51], Uganda [48], and Ethiopia [36,37] found a lack of transport and rising transport fees to be barriers to accessing maternity care during COVID-19. Evidence illustrates how fear of contracting the virus was a barrier to the uptake of maternity care in Sierra Leone [52], Guinea [49], Nigeria [49,50,51], Tanzania [49], Uganda [21,48,49], Kenya [40,42,46], and Ethiopia [36,37] during COVID-19 (Table 1). Similarly, shortages of medical supplies [34,57], a lack of PPE [37,51], shortages of human resources [50], health staff burnout [51], a lack of skilled workers [21], the shift of healthcare providers to COVID-19 centres [59], and closure of non-emergency services [49,59] were identified as barriers to accessing to maternity care during COVID-19 (Table 1).

## 4. Discussion

Over the past two decades in Sub-Saharan African countries, improvements in the quality and availability of ANC and institutional birth have been made, leading to a substantial decline in maternal and neonatal mortality rates. In the 2000–2020 period, remarkable progress was made in these countries in lowering maternal and newborn mortality [65]. Nonetheless, Sub-Saharan countries still experience a significant number of maternal and newborn deaths.

Findings show that in Sub-Saharan countries, ANC utilisation declined during the pandemic [22,31,32,45,49,53,56,59,60,61,63]. This finding coincides with studies conducted in India [66], a systematic review of the impact of the Ebola virus on maternal and perinatal care in West Africa [67], and a worldwide systematic review and meta-analysis which revealed significantly decreased ANC uptake during the pandemic [68]. The decline in ANC utilisation was a consequence of a range of factors such as the lockdowns [66], fear of contracting the virus [69], a lack of transport [70], the shortage of medical supplies [44], and the long waiting times [50].

ANC contact can play a significant role in promoting and increasing institutional birth, which leads to a reduction in the number of neonatal and maternal deaths [71]. ANC achieves this by preventing pregnancy-related complications through early identification and treatment of existing diseases [72]. Conversely, low engagement with ANC can decrease institutional birth rates, leading to increased neonatal and maternal mortality [72].

In this review, some studies demonstrate that the number of institutional births was not significantly altered during the pandemic compared to pre-COVID-19 in Ethiopia [33], DRC [53], and Zimbabwe [62]. This could be because less health facilities may have referred labouring women to hospital due to fear of contracting COVID-19 [22].

This review demonstrates that institutional births [30,32,52,57,60,61,63] substantially decreased during the pandemic. This finding aligns with studies conducted in India [73] and Nepal [25,74], which indicated that institutional birth decreased by half during the COVID-19 lockdowns. This dramatic reduction in institutional births might be due to women’s fears of contracting the virus [21,36,37,40,41,44,46,48,50,51,59], a lack of transport availability during the lockdowns [36,37,44,48,50,60], financial hardship [40,46], and health facilities becoming inaccessible [44,51,59]. Consequently, women might prefer traditional birth attendants’ home support [40,41,57].

Similarly, the findings from this review showed an increase in caesarean section births during the pandemic [31,32,39]. The rise in births by caesarean section could have been due to less maternal and foetal monitoring during labour [25], the restriction on companions attending births [75], and women choosing a caesarean section instead of waiting for spontaneous labour due to fear of contracting the virus [76]. The decline in accessing ANC follow-up can prevent women from receiving early detection of pregnancy complications and health promotion counselling and can lead to an increase in home births [40,41,57] and obstetric complications [48,62]. These findings are consistent with a study conducted in LMICs [77] which explored the decrease in ANC and the rise in home births during the pandemic.

Findings demonstrate that during the pandemic, obstetric complications were more common, potentially leading to an increase in stillbirths [31,32,39] and neonatal deaths [32,47,55,56]. These complications and consequences may have been due to the lower ANC uptake and suboptimal care during the antenatal period that led to unidentified and untreated existing diseases such as preeclampsia, increasing the risk of stillbirth [78] and neonatal deaths [79]. In addition, suboptimal intrapartum care and home birth may have led to increased rates of stillbirth [80] and neonatal deaths [81].

The synthesised evidence emerging from this systematic review of the impact of COVID-19 on maternal health service utilisation in Sub-Saharan Africa has implications for future responses to similar emergencies. From this evidence, policymakers and obstetric care providers can gain insight into how the pandemic in Sub-Saharan Africa has affected the provision and uptake of maternal health services. In health facilities, the existing guidelines need to be adopted and implemented and the modification of maternal and neonatal safety guidelines should be a priority during any pandemic. Furthermore, this review provides an input and lessons learned that can serve as a touchstone for a better understanding of and response to the direct and indirect impacts of future epidemics and pandemics.

Some potential limitations of this review are noted, including the small sample size of some studies, methodological differences, no population-level denominators, missing data, and a lack of population-level data collection methods in place. Moreover, all included studies did not specifically address the consequences of COVID-19 on access, uptake, and provision of ANC and institutional birth. The results of this systematic review should be taken cautiously, given that the included studies represent only a few countries, and full-text language was restricted to English. Nevertheless, the results from this systematic review are a valuable input to designing policies for scaling up the coverage and quality of ANC and institutional birth through interventions that promote adopting and adapting safe maternity care guidelines in present and future pandemics [82,83].

## 5. Conclusions

The findings from this review showed a decrease in the number of women accessing ANC and institutional birth during the COVID-19 pandemic and an increase in births by caesarean section and neonatal deaths. Based on these findings, it is recommended that stakeholders and healthcare providers act to reverse the decline in ANC and institutional uptake by collaborating via community mobilisation/involvement and building trust with the community regarding access to maternal healthcare services. The government needs to ensure the availability of essential medical supplies in hospitals during pandemics, while healthcare providers need to strictly monitor maternal and foetal health during labour to reduce the risk of institutional neonatal deaths. Rigorous studies are needed to examine both the short- and long-term impacts of COVID-19 on maternal and perinatal outcomes.

## Figures and Tables

**Table 1:** Summary of studies included in the systematic review in Sub-Saharan Africa and key findings.

Author, Year, Country	Study Design	Study Population (N)	Study Objective	Outcome Measures	Summary of Findings	QS
Akaba, 2022 [50] Nigeria	Qualitative	IDIs = 54 (pregnant women, healthcare providers, and policymakers)	To explore facilitators and barriers to accessing maternal and child health during the pandemic	Barriers to the uptake of maternal healthcare	Fear of contracting COVID-19, transport difficulties, stigmatisation of sick persons, lack of PPE, lack of medical commodities, long waiting times at hospitals, and shortage of human resources were barriers to accessing maternal healthcare.	8/9
Aranda, 2022 [22] in African countries (Lesotho, Liberia, Malawi, Sierra Leone)	Interrupted time series	All pregnant women who registered in HMIS of 37 health facilities from 2016 to 2021	To assess the impact of COVID-19 on the use of maternal health services	Prevalence of ANC, institutional birth	ANC1 uptake significantly declined by 32% in Sierra Leone during the pandemic. Institutional birth decreased in Lesotho, Liberia, and Sierra Leone.	
Arena, 2023 [54] DR Congo	Retrospective cohort study	N = 14,300 (all women who have given birth and are registered in HMIS)	To compare adverse birth outcomes before and during the pandemic	Prevalence of preterm births, low-birth-weight births, and stillbirths	Around 22% of adverse birth outcomes were reported pre-COVID-19. There was a notable reduction (to 14.3%) in adverse birth outcomes during COVID-19 compared with the pre-COVID-19 period. Preterm birth significantly decreased during COVID-19 compared with before COVID-19 (8.6% vs. 11.5%, *p* < 0001).	9/9
Asuming, 2022 [63] Ghana	Pre–post study design	All pregnant women who have given birth (N = before COVID-19 = 312 and during COVID-19 = 223)	To estimate the impact of COVID-19 on ANC and institutional birth	Prevalence of ANC and institutional birth	Women attending ANC4 more significantly decreased by 25%, and institutional birth significantly decreased by 23% during the pandemic.	
Babalola, 2022 [59] Liberia	Mixed methods	All pregnant women who registered in HMIS and total participants in FGDs = 292	To examine and explore the effects of COVID-19 on maternal healthcare	Prevalence of ANC and barriers to its uptake	Women attending hospitals for ANC1 and ANC4 decreased by 25% and 28% during the pandemic. Institutional birth decreased by 5% during the pandemic. Fear of contracting COVID-19 infection, redeployment of staff to COVID-19 centres, restriction of movement due to lockdowns, lack of PPE, lack of drugs and vaccination supplies for clients, and partial closure of routine services were commonly disturbed maternal healthcare services.	7/9
Banke-Thomas, 2022 [49] in four African countries (Nigeria, Tanzania, Uganda, and Guinea)	Mixed methods	All pregnant women who have given birth and are registered in HMIS and IDIs = 50 (maternity care providers)	To assess the effect of COVID-19 on maternal health service utilisation	Prevalence of ANC, institutional birth, and barriers to uptake it	ANC consultation decreased in Nigeria, Tanzania, Uganda, and Guinea during the first wave of the pandemic. Institutional birth decreased in Tanzania and Nigeria during the first wave of the pandemic. Fear of being infected in hospitals, lack of transport, high transport fees, and service closures affected maternal healthcare services during the first wave of the pandemic.	9/9
Bekele, 2022 [37] Ethiopia	Mixed methods	All pregnant women who have given birth and are registered in HMIS from before COVID-19, March to August 2019, and during COVID-19, March to August 2020, and IDIs = 91 (healthcare providers)	To assess maternal, neonatal and child health service utilisation before and during the first six months of the pandemic	Prevalence of ANC and institutional birth; barriers to accessing maternal and child health services	The utilisation of ANC1, ANC4, and institutional births did not significantly differ before and during COVID-19. Fear of acquiring the virus, travel restrictions, increased cost of transport, lack of PPE, and lack of transport were barriers to the uptake of maternal and child health services.	9/9
Burt, 2021 [47] Uganda	Interrupted time series	All pregnant women who have given birth and are registered in HMIS from July 2019 to March 2020, before COVID-19, and April 2020 to December 2020, during COVID-19	To describe the impact of COVID-19 on maternal, neonatal, and child health outcomes	ANC, institutional birth, neonatal mortality	ANC significantly decreased (*p* = 0.001) during lockdowns. The median number of monthly institutional births was 1869 (IQR 1791–1924) before lockdowns. Early on during the lockdowns, there were 320 institutional births (320, 95% CI; 199 to 441; *p* = 0.0002). There were 109 institutional births per month (109, 95% CI; 55 to 163; *p* = 0.002) during the lockdowns. The median number of monthly institutional births significantly increased by 117 (117, 95% CI 54 to 180; *p* = 0.003) after lockdowns. The low birth rates significantly increased by 70% (1.7, 95% CI: 0.6% to 2.7%; *p* = 0.011) during lockdown. Stillbirth rates increased by 1% (1, 95% CI: −2% to 4%; *p* = 0.58) post-lockdowns. Preterm birth rates increased by 6% (6, 95% CI: −3% to 15%; *p* = 0.22) post-lockdowns. The median number of monthly neonatal admissions to the neonatal intensive care unit (NICU) was 700 (IQR 652–706), and the neonatal death rate was 39.6/1000 livebirths (IQR 34.6–50.7) prior to lockdowns. During the lockdowns, neonatal admission increased by 5.6% (5.6, 95% CI: 0%–11%; *p* = 0.06). The neonatal mortality rate increased by 10 deaths per 1000 live births/month (IQR 2–10; *p* < 0.001) at the end of lockdown.	9/9
Das Neves, 2021 [57] Mozambique	Mixed methods	All pregnant women who have given birth and are registered in HMIS from March to May 2019, before, and March to May 2020, during, COVID-19; IDIs = 19	To assess the impact of COVID-19 and government restrictions on access to maternal and child healthcare services	Prevalence of ANC, institutional birth, caesarean section birth	During COVID-19: ANC1 declined by 19% (*p* = 0.327). Institutional births significantly decreased by 4% (*p* = 0.046). Caesarean sections dropped by 28% during COVID-19 (*p* = 0.135). Home births increased by 74% (*p* = 0.074). Women decreased uptake of services due to fear of contamination and contracting the virus at facilities.	8/9
Desta, 2021 [31] Ethiopia	Pre–post study design	Pregnant women who have given birth and are registered in HMIS (N = 47,896 before COVID-19; N = 50,327 during COVID-19)	To demonstrate the impact of COVID-19 on essential health services	Pregnancy complications, mode of birth, maternal mortality, pregnancy loss, neonatal mortality	During COVID-19: ANC4 decreased by 2.83% (*p* = 0.5761). ANC1 slightly increased by 5.08% (*p* = 0.0978). Institutional births increased by 8.57% (*p* = 0.0001). Caesarean births increased by 28.05% (*p* = 0.0040). The stillbirth rates also increased by 18.57% (*p* = 0.0062). Institutional maternal deaths slightly decreased by 17% (*p* = 0.3173), and institutional neonatal death slightly decreased by 46.81 (*p* = 0.0733).	8/9
Enbiale, 2021 [33] Ethiopia	Comparative cross-sectional study	All pregnant women who have given birth and are registered in HMIS	To assess the effect of preventive COVID-19 measures on essential healthcare services	Prevalence of ANC and institutional birth	ANC and institutional birth did not significantly decrease during COVID-19.	6/9
Gebreegziabher, 2022 [35] Ethiopia	Cross-sectional data review	All pregnant women who have given birth and are registered in HMIS	To evaluate the impact of COVID-19 on maternal and child health services	Trends of ANC, institutional birth, and PNC uptake	Monthly ANC follow-up and institutional birth did not significantly differ before and during the pandemic.	9/9
Hailemariam, 2021 [34] Ethiopia	Qualitative	FGDs = 44 pregnant women; IDIs = 9 health providers	To explore COVID-19-related factors influencing ANC service uptake in rural Ethiopia	Barriers to ANC utilisation	Poor quality of care; shortage of logistic supplies; and decreased staff motivation due to lack of risk allowance and accommodations, and increased anxiety related to infections were barriers to the uptake of ANC during the pandemic.	7/9
Hategeka, 2021 [53] DR Congo	Interrupted time series	All pregnant women who have given birth and are registered in HMIS	To assess the utilisation of ANC1, the number of institutional births, and PNC2 before and during the national COVID-19 lockdown in DR Congo	ANC1 utilisation and institutional birth before and during COVID-19 and lockdowns	At the start of COVID-19 lockdowns: Institutional birth trends (*p* = 0.51) did not significantly decrease. Utilisation of ANC1 decreased by 43% during lockdowns (IRR: 0.57, 95%CI: 0.35 to 0.91, *p* = 0.02). Immediately post-lockdowns: ANC1 contact significantly increased by 4% (1.04, 95% CI: 1.01 to 1.07, *p* = 0.007). Institutional birth significantly increased by 8% (1.08, 95% CI: 1.05 to 1.011, <0.001).	9/9
Jensen, 2020 [55] South Africa	Retrospective review of the District Health Information System	All neonates who registered in HMIS	To assess the impact of local COVID-19 upon routine child health services	The rate of neonatal death	Institutional neonatal death significantly increased by 47% during COVID-19 (*p* = 0.025).	7/9
Kassie, 2021 [32] Ethiopia	Comparative cross-sectional study	Pregnant women who have given birth and are registered in HMIS (N = 3773 before COVID-19; N = 2739 during COVID-19)	To estimate the impact of COVID-19 on the utilisation of reproductive, maternal, and neonatal health services	ANC, institutional birth, caesarean section birth, stillbirth, neonatal intensive care admission, neonatal deaths, and PNC	ANC1 (*p* = 0.0001) and institutional birth (*p* = 0.001) significantly decreased during COVID-19. Teenage pregnancy (*p* = 0.0001), caesarean section births (*p* = 0.0001), stillbirths (*p* = 0.0001), neonatal intensive care admission (*p* = 0.0001), and neonatal deaths (*p* = 0.0001) significantly increased during COVID-19.	7/9
Kayiga, 2021 [48] Uganda	Qualitative	IDIs = 25 (healthcare providers)	To explore healthcare providers’ experiences and perceptions of maternal and neonatal health services during the pandemic	To explore barriers to the provision of maternal healthcare	Fear of contracting COVID-19, lack of transport, and burnout hindered the provision of maternal healthcare.	9/9
Kiarie, 2022 [43] Kenya	Interrupted time series	All pregnant women who have given birth and are registered in HMIS	To assess the effect of COVID-19 on essential healthcare services	Prevalence of ANC, institutional birth	ANC and institutional birth did not significantly differ before and during COVID-19.	9/9
Kouyate, 2022 [60] Guinea	Cross-sectional	All pregnant women who have given birth and are registered in HMIS	To estimate the impact of COVID-19 on ANC and institutional birth	Prevalence of ANC and institutional birth	During COVID-19: Women attending associative health centres (β = −702; 95%CI = −885 to−520; *p* = 0.001) and health centres (β = −64; 95%CI = −137 to 9; *p* = 0.082) for ANC1 significantly declined. Women attending associative health centres (β = −1015; 95% CI = −1146 to −883; *p* = 0.001) and health centres (β = −794; 95% CI = −909 to 678; *p* = 0.001) for ANC4 significantly declined. Institutional births significantly decreased at associative health centres (β = −596; 95% CI = −677 to −516; *p* = 0.001).	
Landrian, 2022 [44] Kenya	Cross-sectional	Women who gave birth before COVID-19 = 1189 and women who gave birth during COVID-19 = 540	To assess the effect of COVID-19 on ANC utilisation	Prevalence of ANC	Women who gave birth during COVID-19 had higher odds of delayed ANC initiation than those who gave birth before the pandemic. The factors hindering early initiation of ANC were closed facilities, fear of contracting the virus, movement restrictions, inability to pay for or lack of transport, and inability to afford care.	9/9
Leung, 2022 [51] Nigeria	Qualitative	IDIs = 16 (maternity care providers)	To explore the perception and experiences of maternity care workers on maternal healthcare during COVID-19	Barriers to the provision of maternal healthcare	Fear of infection, burnout, transport difficulties, inadequate PPE, poverty, lockdown, and health system weakness were barriers to accessing maternity care.	8/9
Lusambili, 2020 [40] Kenya	Qualitative	IDIs = 25 (pregnant women and maternity care providers	To explore the impact of COVID-19 on women refugees’ access to and utilisation of ANC, birth, and PNC	Barriers to utilisation of ANC, birth, and PNC during COVID-19	Women living in refugee communities increasingly preferred to give birth at home during COVID-19. Delayed care, fear, economic hardship, and decreased facility-based births were barriers to utilisation of services by refugee women.	7/9
Lydon, 2022 [58] Mozambique	Interrupted time series	All pregnant women who have given birth and are registered in HMIS	To assess the effect of COVID-19 on maternal and perinatal health service utilisations and outcomes	Prevalence of ANC, institutional birth, caesarean section birth, and its outcomes	ANC1 contact increased by 29.8% (95% CI 18.2 to 41.4%) per month before COVID-19. ANC4 contact did not significantly differ between before and during COVID-19, a difference of 0.5% (95% CI −8.8 to 9.9 (*p* = 0.91). Institutional births increased by 6.1% (95% CI 0.03 to 12.2%) per month compared with before COVID-19. Caesarean births decreased by 30.1% (95% CI −55.0 to −5.3%) per month compared with before COVID-19. Uterine ruptures decreased by 5.3% (95% CI −9.9 to −0.6%) per month compared with before COVID-19. Stillbirths decreased by 19.2% (95% CI −33.8 to −4.6%) per month compared with before COVID-19.	9/9
Nakate, 2022 [21] Uganda	Qualitative	IDIs = 14 (pregnant and postnatal women)	To explore women’s experiences in the first 1000 days post conception during the pandemic	To explore barriers to the uptake of maternal healthcare	Distress situations, living in fear, making forced choices, and a lack of access to expert care were barriers to the uptake of maternal healthcare during the pandemic.	9/9
Oluoch-Aridi, 2020 [46] Kenya	Qualitative	IDIs = 71 (postnatal women)	To investigate the effect of COVID-19 on access to maternal healthcare services in the informal settlement	To explore barriers to accessing maternal healthcare	Fear of infection and economic and food security challenges hindered access to healthcare during COVID-19.	7/9
Ombere, 2021 [41] Kenya	Qualitative	IDIs = 21 (pregnant and postnatal women, and maternity care providers)	To explore the effect of COVID-19 on maternal service utilisation	Barriers to utilisation of maternal services during COVID-19	Pregnant women decreased their attendance at hospitals for perinatal care and institutional birth due to fear of infection. Home births and births assisted by a traditional birth attendant increased during COVID-19. PPE was lacking.	7/9
Onchonga, 2021 [42] Kenya	Qualitative	FGDs = 4; N = 26 (pregnant women)	To explore women’s understanding of health-seeking during the pandemic	Barriers to women’s health-seeking during COVID-19	Fear of contracting the virus was the main factor hindering maternal health uptake during the pandemic.	7/9
Pillay, 2021 [56] South Africa	Retrospective review of the District Health Information System	All pregnant women who have given birth and are registered in HMIS	To determine the effect of COVID-19 and restrictions imposed on routine health services	Prevalence of ANC, institutional birth, neonatal death, and maternal deaths	ANC uptake significantly decreased during COVID-19. Institutional birth increased during COVID-19. Institutional neonatal and maternal mortality increased during COVID-19.	
Quaglio, 2022 [38] four African countries	Interrupted time series	All pregnant women who have given birth and are registered in HMIS	To examine the indirect effect of COVID-19 on maternal health services utilisation	Prevalence of ANC and institutional birth	Monthly ANC visits (*p* = 0.71) and institutional birth (*p* = 0.14) did not significantly increase during COVID-19.	8/9
Sevalie, 2021 [52] Sierra Leone	Mixed methods	All pregnant women who have given birth and are registered in HMIS and IDIs = 12 (service users and maternity care providers)	To examine and explore the effects of COVID-19 on hospital utilisation	Facility-based birth, caesarean birth, and barriers to uptake in maternal healthcare	Institutional birth significantly increased from 435 to 467, a 7.5% increase (*p* = 0.033) from the first quarter (Q1) to the second quarter; however, institutional birth significantly decreased by 10% from 435 Q1 388 in Q3 (*p* = 0.007) during COVID-19. Caesarean section births significantly increased from 192 to 216, a 12.7% increase (*p* = 0.014) from the first quarter to the second quarter. During COVID-19, there was no change in caesarean section births from Q1 to Q3. Decreased non-emergency services, delays in giving care, increased staff load, fear of contracting the virus, and loss of income were barriers to the uptake of maternal healthcare.	9/9
Shakespeare, 2021 [62] Zimbabwe	Retrospective observational study	All pregnant women who have given birth and are registered in HMIS	To examine the impact of COVID-19 on maternal and perinatal care and outcomes during the lockdown	ANC, institutional birth, caesarean section birth, stillbirth, and maternal mortality ratio	The mean monthly births reduced from 747 (SD ± 61.3) to 681 (SD ± 17.6) (*p* = 0.2) during lockdowns. The percentage of institutional births booked by women in the hospital dropped from a mean of 41.6% (SD ± 1.1) to 35.8% (SD ± 4.3) (*p* = 0.03) during lockdowns. The percentage of institutional births unhooked by women in the hospital increased from a mean of 4.4% (SD ± 0.6) to 8.0% (SD ± 2.5) (*p* = 0.01) during lockdowns. The rate of caesarean birth before COVID-19 was 29.8% (SD ± 1.7) versus 28.0% (SD ± 1.7) during COVID-19 (*p* = 0.18). The mean total number of early neonatal deaths (ENND) increased (mean 18.7 (SD ± 2.9) versus 24.0 (SD ± 4.6) (*p* = 0.32)) during lockdowns.	9/9
Shikuku, 2021 [39] Kenya	Cross-sectional	All pregnant women who have given birth and are registered in HMIS	To assess the initial impact of the pandemic on reproductive, maternal, newborn, child, and adolescent health services	Prevalence of ANC, institutional birth, caesarean section birth, stillbirth, and maternal mortality ratio	Monthly utilisation of ANC (*p* = 0.251) and institutional birth (*p* = 0.736) did not significantly differ before and during COVID-19. Adolescent pregnancy (*p* = 0.0001), caesarean section birth (*p* = 0.0001), and stillbirth (*p* = 0.0066) significantly increased during COVID-19. The maternal mortality ratio did not significantly differ before and during COVID-19 (*p* = 0.1023).	6/9
Tilahun, 2022 [36] Ethiopia	Mixed methods	All pregnant women who have given birth and are registered in HMIS and IDIs = 74 (women and healthcare providers)	To explore and examine the effect of COVID-19 on maternal and child health services	Prevalence of ANC and institutional birth; barriers to accessing maternal and child health services	Utilisation rates of ANC1, ANC4, and institutional birth did not significantly differ before and during COVID-19. Fear of contracting COVID-19, imposed movement restriction, increased workload, and shortage of PPE were hindrances to accessing maternal and child health services.	8/9
Wambua, 2022 [45] Kenya	Interrupted time series	All pregnant women who have given birth and are registered in HMIS	To quantify the indirect effect of the pandemic on the utilisation of outpatient services	Prevalence of ANC	ANC4 significantly decreased during the pandemic. However, ANC1 did not significantly differ before and during the pandemic.	9/9
Wanyana, 2021 [61] Rwanda	Cross-sectional	Pregnant women: N = 59,810, expected number before COVID-19; N = 61,205, expected number during COVID-19	To assess the change in utilising maternal and child health (MCH) services during the COVID-19 outbreak	ANC, institutional birth	ANC1 (*p* = 0.042) and institutional birth (*p* = 0.004) significantly declined during COVID-19. ANC4 (*p* = 0.083) did not significantly differ before and during the pandemic.	9/9
Zimmerman, 2021 [30] Ethiopia	Pre–post study design	Pregnant women N = 2537	To examine the effect of COVID-19 on health facility birth	Institutional birth	Institutional birth in the hospital decreased by 77% in urban areas during COVID-19 (aRRR: 0.23, 95% CI: 0.07–0.71).	9/9

Abbreviations: ANC: antenatal care; ANC1: first antenatal care visit (booked visit); ANC4: fourth antenatal care visit; FGDs focus group discussions; HMIS: hospital management information system; IDIs: in-depth interviews; IRR: incidence rate ratio; PPE: personal protective equipment; QS: quality score.

**Table 2:** Main themes.

Themes	Study Authors
Decrease in the number of women attending ANC	[22,31,32,33,34,35,36,37,38,39,41,43,44,45,47,48,49,52,53,56,57,58,59,60,61,62,63]
Impact of COVID-19 on institutional birth and mode of birth	[22,30,31,32,33,35,36,37,38,39,41,43,47,52,53,56,57,58,59,60,61,62,63]
Complications during childbirth	[31,32,39,40,41,47,52,54,55,56,57,58,62]
Barriers to the uptake of maternity care	[21,34,36,37,40,41,42,44,46,48,49,50,51,52,57,59]

## Data Availability

All analysed data are included in this manuscript. PROSPERO registered number = CRD42020208198.

## Supplementary Materials

The following supporting information can be downloaded at: https://www.mdpi.com/article/10.3390/ijerph21091188/s1, Preferred Reporting Items for Systematic Reviews and Meta-Analysis (PRISMA) checklist [84].

## Abbreviations

ANC = antenatal care, EVD = Ebola virus disease, MERS = Middle East respiratory syndrome, NC = postnatal care, SARS = severe acute respiratory syndrome, SDG = Sustainable Development Goal, SARS-CoV-2 = severe acute respiratory syndrome coronavirus 2, and WHO = World Health Organization.
